# Threats to intact tropical peatlands and opportunities for their conservation

**DOI:** 10.1111/cobi.12925

**Published:** 2017-07-10

**Authors:** K.H. Roucoux, I.T. Lawson, T.R. Baker, D. Del Castillo Torres, F.C. Draper, O. Lähteenoja, M.P. Gilmore, E.N. Honorio Coronado, T.J. Kelly, E.T.A. Mitchard, C.F. Vriesendorp

**Affiliations:** ^1^ School of Geography and Sustainable Development University of St Andrews Irvine Building, North Street St Andrews Fife KY16 9AL U.K.; ^2^ School of Geography University of Leeds Leeds LS6 9JT U.K.; ^3^ Instituto de Investigaciones de la Amazonía Peruana Iquitos, Av. José A. Quiñones km. 2.5 ‐ Apartado Postal 784 Loreto Peru; ^4^ Department of Global Ecology Carnegie Institution for Science 260 Panama St Stanford CA 94305 U.S.A.; ^5^ International Center for Tropical Botany Florida International University 11200 SW 8th St Miami FL 33199 U.S.A.; ^6^ School of Life Sciences Arizona State University Tempe AZ 85287 U.S.A.; ^7^ School of Integrative Studies George Mason University Fairfax VA 22030 U.S.A.; ^8^ School of GeoSciences,The University of Edinburgh Crew Building,The King's Buildings Edinburgh EH9 3FF U.K.; ^9^ Science and Education The Field Museum of Natural History 1400 S Lake Shore Dr Chicago IL 60605 U.S.A.

**Keywords:** Amazonia, carbon, peatland, Peru, peat, tropics, Amazonía, carbono, trópicos, turba, turberas

## Abstract

Large, intact areas of tropical peatland are highly threatened at a global scale by the expansion of commercial agriculture and other forms of economic development. Conserving peatlands on a landscape scale, with their hydrology intact, is of international conservation importance to preserve their distinctive biodiversity and ecosystem services and maintain their resilience to future environmental change. We explored threats to and opportunities for conserving remaining intact tropical peatlands; thus, we excluded peatlands of Indonesia and Malaysia, where extensive deforestation, drainage, and conversion to plantations means conservation in this region can protect only small fragments of the original ecosystem. We focused on a case study, the Pastaza‐Marañón Foreland Basin (PMFB) in Peru, which is among the largest known intact tropical peatland landscapes in the world and is representative of peatland vulnerability. Maintenance of the hydrological conditions critical for carbon storage and ecosystem function of peatlands is, in the PMFB, primarily threatened by expansion of commercial agriculture linked to new transport infrastructure that is facilitating access to remote areas. There remain opportunities in the PMFB and elsewhere to develop alternative, more sustainable land‐use practices. Although some of the peatlands in the PMFB fall within existing legally protected areas, this protection does not include the most carbon‐dense (domed pole forest) areas. New carbon‐based conservation instruments (e.g., REDD+, Green Climate Fund), developing markets for sustainable peatland products, transferring land title to local communities, and expanding protected areas offer pathways to increased protection for intact tropical peatlands in Amazonia and elsewhere, such as those in New Guinea and Central Africa which remain, for the moment, broadly beyond the frontier of commercial development.

## Abstract (es)

Amenazas a las Turberas Tropicales Intactas y las Oportunidades para su Conservación

**Resumen**

Extensas e intactas áreas de turberas tropicales están severamente amenazadas a escala mundial, debido a la expansión de la agricultura comercial y a otras formas de desarrollo económico. La conservación de las turberas como parte del medio natural, con su hidrología no intervenida, es de importancia internacional para asegurar la conservación de su biodiversidad tan característica y de los servicios ambientales que ofrecen, y así mantener su resiliencia ante futuros cambios ambientales. Exploramos las amenazas y las oportunidades para conservar las turberas tropicales intactas y remanentes. Nuestro enfoque por lo tanto excluye las turberas de Indonesia y Malasia, donde la deforestación de forma extensiva y el drenado para el establecimiento de plantaciones hace que la conservación en estas regiones pueda proteger solo pequeños fragmentos del ecosistema original. En este estudio, nos enfocamos en las cuencas del Pastaza‐Marañón (PMFB, siglas en inglés) en Perú, que comprende una de las turberas tropicales intactas más extensas del mundo y es representativa de la vulnerabilidad que las amenaza. El mantenimiento de las condiciones hidrológicas críticas para el almacenamiento de carbono y la función ambiental de las turberas está, en la PMFB, amenazada principalmente por la expansión de la agricultura comercial y las nuevas vías de transporte que facilitan el acceso a las áreas remotas. En la PMFB y en otros lugares aún hay oportunidades para desarrollar prácticas alternativas y sostenibles del uso del suelo. Aunque algunas de las turberas en la PMFB caen dentro de áreas naturales protegidas, esta protección no incluye las áreas con mayor densidad de carbono (varillal hidromórfico). Nuevas herramientas para la conservación basada en el carbono (p.e. REDD+, Fondo Verde para el Clima), el desarrollo de mercados para los productos de las turberas, la transferencia de títulos de propiedad de la tierra a las comunidades locales, y la expansión de las áreas protegidas ofrecen alternativas para una mayor protección de las turberas tropicales intactas en la Amazonía y en otros lugares, como las turberas de Nueva Guinea y África Central que permanecen, por el momento, alejadas de la frontera de desarrollo comercial.

## Introduction

Catastrophic fires in Indonesia in late 2015 (Chisholm et al. 2016) represent just the latest episode in the destruction and degradation of some of the most extensive peatland ecosystems in the tropics. The fires highlighted a now well‐documented failure to protect these carbon‐dense systems from the combined effects of land‐use change, drainage, and episodic El Niño droughts that lead to loss of not only habitats but also release of belowground carbon (BGC). Fortunately, in Amazonia, Africa, and New Guinea tropical peatland ecosystems are also widespread and often much less intensively exploited. Many can be described as intact at the landscape scale; their hydrology is unaffected by human activity and their vegetation cover is not fragmented or substantially degraded. However, their importance is weakly articulated within existing conservation agendas, principally because they are poorly described and mapped and are frequently unrecognized by local agencies and institutions. We examined the services provided by large, intact tropical peatlands, factors threatening them, and opportunities to conserve them.

**Figure 1:**
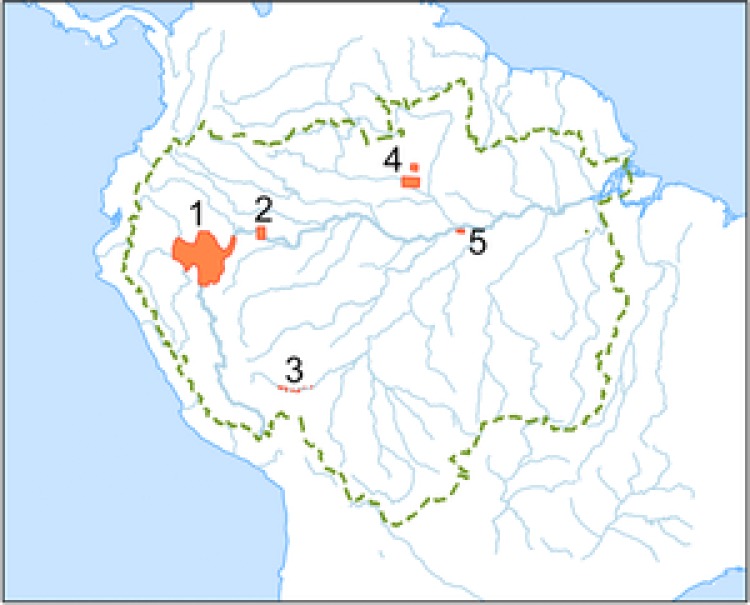
Outline of the Amazon Basin (dashed line), major rivers (solid lines), and peatland areas (shaded): 1, Pastaza‐Marañón Foreland Basin; 2, floodplains of the Amazon River and its tributaries; 3, Madre de Díos; 4, Rio Negro basin; 5, Negro and Solimões confluence. Sources of mapped areas are in Supporting Information.

We drew most of our examples from the Pastaza‐Marañón Foreland Basin (PMFB) in Peruvian lowland Amazonia (Fig. 1) because the large extent and high carbon density of its peatlands, unrecognized until recently, have stimulated scientific research and a reappraisal of conservation strategies that have potential application elsewhere. The existence of peat deposits in this part of the Amazon Basin became known in the early 2000s (Schulman et al. 1999; Ruokolainen et al. 2001; Freitas et al. 2006), and the first systematic study of their thickness and extent was by Lähteenoja et al. (2009a, b). Up to 7.5 m of peat has accumulated over the last 8900 years (Lähteenoja et al. 2012) and covers about 3.5 million ha (Draper et al. 2014). Current best estimates of the distribution of peat across the tropics (Page et al. 2011), including extensive new discoveries in the Cuvette Centrale of the Congo Basin (Dargie et al. 2017), suggest that the approximately 3 Gt C stored in the PMFB (Draper et al. 2014) represents about 2.7% of the tropical peatland carbon stock. Carbon storage in peatlands is closely tied to waterlogging, which encourages the anaerobic conditions that limit the decomposition of organic matter. The drainage required to convert peatlands for other uses, such as oil‐palm plantations, typically leads to rapid aerobic peat decomposition and loss of carbon to the atmosphere, in addition to the aboveground carbon (AGC) and biodiversity losses experienced by other forest ecosystems. Peatlands are therefore vulnerable to human disturbance and degradation in qualitatively different ways from other forest ecosystems, and the resulting carbon emissions per area may be disproportionately large.

**Figure 2:**
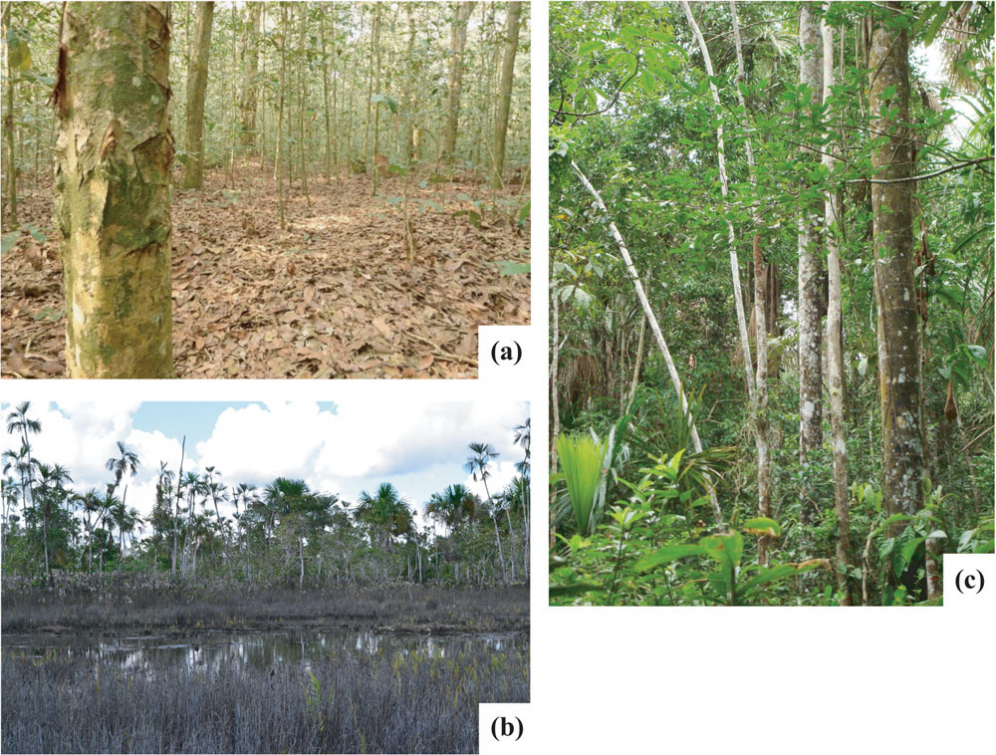
Examples of the 3 main peatland vegetation types in the Pastaza‐Marañón Foreland Basin, Peru: (a) pole forest, (b) open peatland, and (c) palm swamp.

**Figure 3:**
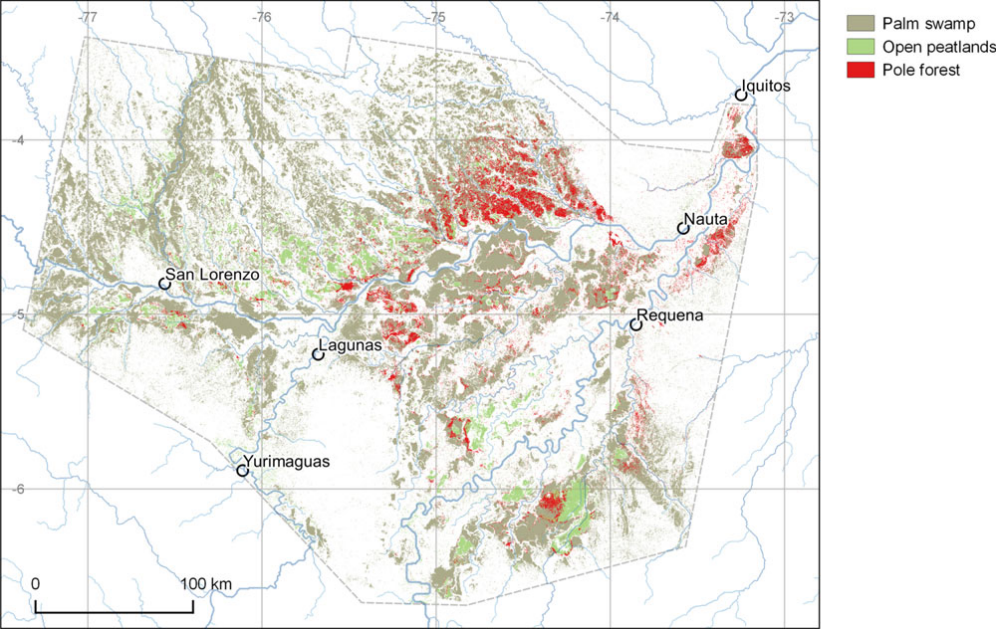
Modeled distribution of palm swamp, pole forest, and open peatland vegetation in the Pastaza‐Marañón Foreland Basin, Peru, based on field and remote‐sensing data (Draper et al. 2014) (see Supporting Information for cartographic data sources).

Similar to their counterparts in Southeast Asia, peatlands in the PMFB are composed of a range of vegetation types (Figs. 2 & 3) (Draper et al. 2014). The most widespread type is palm swamp (characterized by abundant *Mauritia flexuosa* palms), which covers 2.8 million ha. Peatland pole forest (characterized by short, thin‐stemmed trees) covers 350,000 ha, is apparently restricted to wholly rain‐fed, nutrient‐poor peat domes, and is similar in structure to forests on ombrotropic peatlands in Southeast Asia (Anderson 1983). Peatland pole forest is the most carbon‐dense ecosystem type in Amazonia; 1391 Mg C/ha (SE 710) is stored, mostly belowground (Draper et al. 2014). The third vegetation, open peatland (410,000 ha), is dominated by herbaceous communities that have not been described in detail.

Compared with the hyperdiverse terra firme forests in the region, the peatland vegetation types have low stand‐scale (*α*) diversity. However, they are floristically distinctive because they are dominated by a small number of specialist species, although they do have a subset of the species common to terra firme forests (Draper 2015). Their compositional distinctiveness likely reflects a physiologically demanding environment (anoxic, waterlogged, and in the case of peat domes, nutrient‐deficient substrate [Rydin & Jeglum 2013]) and the effects of disturbance on centennial to millennial time scales through river‐channel migration (Roucoux et al. 2013).

The human population density in the PMFB is low (2.4 people/km^2^ in Loreto [INEI 2015]), but the basin is not an untouched wilderness. Palm swamps are recognized by local forest communities as being resource rich and are used for hunting game and harvesting plant resources. Over 50 plant species are used for construction, food, medicine, and ceremonial purposes, and several forest products enter the formal economy (e.g., *M. flexuosa* fruits and vanilla orchid [Householder et al. 2010; Gilmore et al. 2013]).

The PMFB peatlands are also important habitats for animals. In palm swamps, *M. flexuosa* produces abundant and nutritious fruits that support a diverse and dense fauna, including monkeys, tapirs (*Tapirus terrestris*), peccaries (*Tayassu pecari*), agoutis (*Agouti paca*), macaws (e.g., *Ara* sp.), turtles, and fishes (Gilmore et al. 2013). Pole forest supports several threatened and endangered bird species reported only in nearby forests with similarly nutrient‐poor white‐sand soils (Lähteenoja et al. 2009b). The emerging picture is of a distinctive and specialist flora and fauna of similar value to the charismatic biodiversity of Southeast Asian peatlands (Wich et al. 2016).

## Potential Threats to Tropical Peatland Ecosystems

### Transport Infrastructure

**Figure 4:**
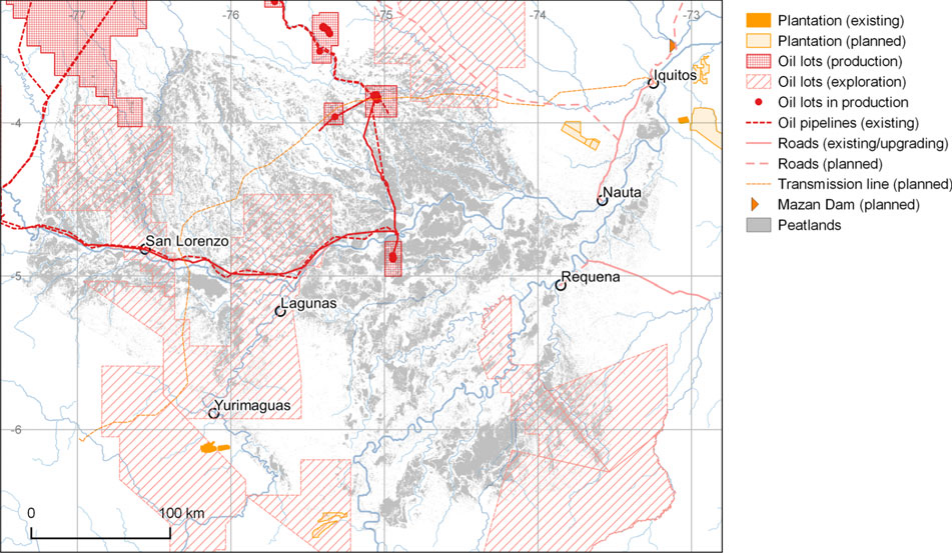
Locations of potential threats to carbon storage and biodiversity in the peatlands of the Pastaza‐Marañón Foreland Basin, Peru (see Supporting Information for sources).

Development of transport infrastructure in tropical forests typically accelerates forest degradation and deforestation (Laurance et al. 2009). The impact is particularly severe where such activities make a first cut through previously largely undisturbed forest (Laurance et al. 2015), a typical scenario for intact tropical peatland landscapes. For example, in the PMFB there are currently few roads and no railroads, and most people and goods travel by river (Figure 4). However, several major infrastructure developments are planned, including the first all‐weather roads to link the PMFB to the rest of Peru and to Brazil and Colombia; improvements to the navigability and port facilities of major river‐transport routes (hidrovías); and an electricity transmission line and service track between Moyobamba and the regional capital, Iquitos (Dourojeanni 2016; La Region 2016). Some of the planned routes pass directly through regions where peatland pole forests are concentrated (Fig. 4).

In the PMFB such new infrastructure may have a transformative effect by reducing transport costs and encouraging investment by commercial agriculture. Improved access to markets for previously isolated villages, towns, and communities also encourages immigration and smallholder agricultural expansion. For example, in better‐connected regions of the Amazon, immigration is substantial (e.g., 6%/year in Madre de Dios, southern Peru), and smallhold farmers have tapped into markets and used migrant labor to increase the area of land under cultivation (Ichikawa et al. 2014). Building roads or railroads through peatlands is also likely to alter drainage networks and hydrological connectivity and thus increase the potential for acute, negative consequences for water‐table levels, drainage paths, and flooding regimes (Barry et al. 1992), which are critical to peatland integrity and carbon‐storage function.

### Agriculture

Expansion of oil‐palm plantations has been a key driver of deforestation of tropical peatlands in Indonesia and Malaysia, and this model of economic development is now expanding to previously intact forest landscapes in other regions. For example, commercial plantations are expanding rapidly in western Amazonia (Gutiérrez‐Vélez et al. 2011) (Fig. 4) as demand for palm oil in particular is growing and new cultivars are making cultivation more profitable (Villela et al. 2014). In the Ucayali Region of Peru, >9400 ha of mostly primary terra firme forest has been cleared for oil‐palm plantation since 2011 (Erickson‐Davis 2015). Immediately east of the PMFB, 2126 ha of primary forest was cleared for cacao and oil palm between May 2013 and August 2014 by United Cacao; the company owns more land nearby and further expansion seems likely (Finer & Novoa 2015). Commercial agriculture has not yet expanded into the peatlands in the PMFB, but plantations and rice paddies have encroached on wetlands and palm swamps elsewhere in Peru (e.g., Madre de Dios [Janovec et al. 2013]) and in Colombia (Potter 2015).

Experience in Indonesia and Malaysia vividly demonstrates the effect of conversion of peatlands to agriculture. Typically, oil‐palm plantations in this region are established on nutrient‐poor peat domes. Drainage causes the entire peat body to undergo enhanced decay, compaction, and oxidation (Moore et al. 2013). The dried‐out surface peats ignite easily, and the ensuing fires can release globally significant quantities of carbon, typically 0.2 Gt C/year in recent decades and up to 0.7 Gt C/year in El Niño years such as 1997 (Dommain et al. 2014). Hergoualc'h and Verchot (2011) conservatively estimate the loss of carbon at 427.2 Mg C/ha (SE 90.7) over the first 25‐year oil‐palm rotation cycle.

Of course, for as long as expansion of plantations in terra firme forest is possible, conversion of peatlands is comparatively unattractive because of the costs of drainage and fertilization and the lack of a valuable timber harvest during clearance. However, any strengthening of the protection for primary terra firme forest without concomitant improvements in protection for peatlands would increase the risk of agricultural expansion into these (currently) less viable areas. The severity of the potential environmental impact provides strong justification for establishing an effective legal barrier to agricultural expansion onto peatlands. In Peru all proposals for development, including plantations, are required to undergo a formal environmental impact assessment, which should mitigate the threat to peatlands as long as their BGC is recognized. However, such legal protection is not always effective: some plantations are alleged to have been established illegally and without proper environmental impact assessment (EIA 2015). In such cases the courts can intervene, although sometimes only after the damage has been done (EIA 2015; USAID 2015). Legal oil‐palm plantations can also have considerable environmental impact. For example, in a worrying legal precedent, policy intended to promote biofuel production has been used to justify and obtain permission to establish plantations in primary forest in San Martín, Peru (Potter 2015), including in palm swamps (D.d.C.T., personal observation).

### Smallholder Activities

Smallholder activity represents an important land use within and around remaining intact tropical peatlands. In the PMFB, cultivation by smallholders is restricted to silty alluvial soils, but palm swamps (including those on peat) are frequently visited by smallholders collecting wild plant resources and hunting game. Ecological impacts can be considerable at sites with easy access to the main regional market in Iquitos. Unsustainable forms of *M. flexuosa* fruit harvesting involving felling female fruit‐bearing trees (Gilmore et al. 2013) and charcoal production (Arce‐Nazario 2007) are widely practiced. In Iquitos, approximately 130 t of *M. flexuosa* fruits are consumed each month that are the product of approximately 1078 trees (the majority of which have been felled to harvest the fruit). This market provides a livelihood for about 4700 people (Rojas et al. 2001; Delgado et al. 2007). These activities directly affect vegetation communities by, for example, changing the male : female sex ratio of *M. flexuosa* populations (Horn et al. 2012) and encouraging increases in the relative abundance of dicotyledonous trees at the expense of palms (Endress et al. 2013). It is unknown whether peatland hydrology is directly affected. Nevertheless, at their present extent such smallholder impacts are unlikely to be a major driver of peatland degradation. However, the growth of commercial plantations in the region is likely to stimulate smallholder participation in cash‐crop (e.g., oil palm) economies, as has happened elsewhere (Sayer et al. 2012), which may extend smallholder impact. Thus, although at present the risk of widespread peatland degradation from commercial and small‐holder agriculture appears to be low, whether this will remain so in future is uncertain.

### Climate Change

The principal threat to peatlands associated with climate change is increased duration or severity of droughts which, by lowering the water table, would lead to rapid degradation of peat through oxidation and combustion (Turetsky et al. 2015). Paleoecological records suggest peat accumulation is sensitive to climate change. For example, a record from the San Jorge domed peatland in the PMFB indicates a pronounced reduction in peat accumulation rate from AD 650 to 1550 that appears consistent with climatic drying (Kelly et al. 2017). Although predictions of future climatic change in remote areas of intact tropical peatlands are uncertain, the trajectory is likely to vary between regions. For example, for western Amazonia, climate models typically predict higher precipitation, greater maximum levels of river discharge (Sorribas et al. 2016; Zulkafli et al. 2016), and less severe and fewer droughts as this century progresses (e.g., Marengo et al. 2012; Langerwische et al. 2013; Sánchez et al., 2015). With temperature increases of 2—4 °C, the overall effects on the precipitation : evaporation ratio would approximately cancel out (Marengo et al. 2012). Hence, in terms of their water balance at least, peatlands in the PMFB may escape the worst effects of 21st century climate change, in contrast to Southeast Asian peatlands, which are strongly influenced by El Niño droughts. This comparatively low risk of increased drought in the PMFB enhances the value of this region as a long‐term carbon store that is worthy of protection.

### Mining, Oil, and Gas

Mineral resource and hydrocarbon extraction can cause substantial deforestation both directly and indirectly. In the PMFB, geological hydrocarbon exploration and extraction are widespread and active (Finer et al. 2015), and some 24% of the modeled peatland area in the PMFB lies within designated oil‐extraction or exploration lots, which also substantially overlap reserves and land held by indigenous communities. Sizeable installations in 3 discrete areas have been operated by PetroPeru since 1972 (Fig. 4). The activities of most concern in relation to hydrocarbon exploitation are the cutting of access roads, pipeline access paths, and seismic survey lines. Most survey lines quickly become overgrown, but some are kept open by local communities. Pipeline routes are routinely kept clear for maintenance purposes, which improves access to remote areas and may affect peatland hydrology if paved. Oil spills are common along pipelines (e.g., La Republica 2013), but their ecological impacts on peatlands have not been studied.

### Hydropower

Hydroelectricity is central to development plans in Amazonia, and the impact of these developments on associated aquatic and terrestrial biodiversity is a major conservation concern (Lees et al. 2016). In western Amazonia, approximately 150 major dams have been planned, including the Mazan Dam just downstream of Iquitos. The largest planned dam is the 4500 MW Manseriche megadam, just upstream of the PMFB on the Rio Marañón (Finer & Jenkins 2012). The consequences for peatlands downstream of new dams have not been studied, but relevant impacts may include changes to the amount of suspended sediment, nutrients, and organic matter transported by rivers and changes to river flow regimes (e.g., reduction of peak flow) (Ligon et al. 1995). Such changes could affect the nutrient status and hydrology of floodplain peatlands and the drainage characteristics of peats on interfluves via changes in river‐water base levels. The interaction between climate change, which may increase river flows, and the development of hydroelectric power schemes, which act to dampen seasonal cycles, presents an added layer of complexity to the effects of hydropower development on the PMFB.

## Opportunities for Conserving Intact Tropical Peatlands

**Figure 5:**
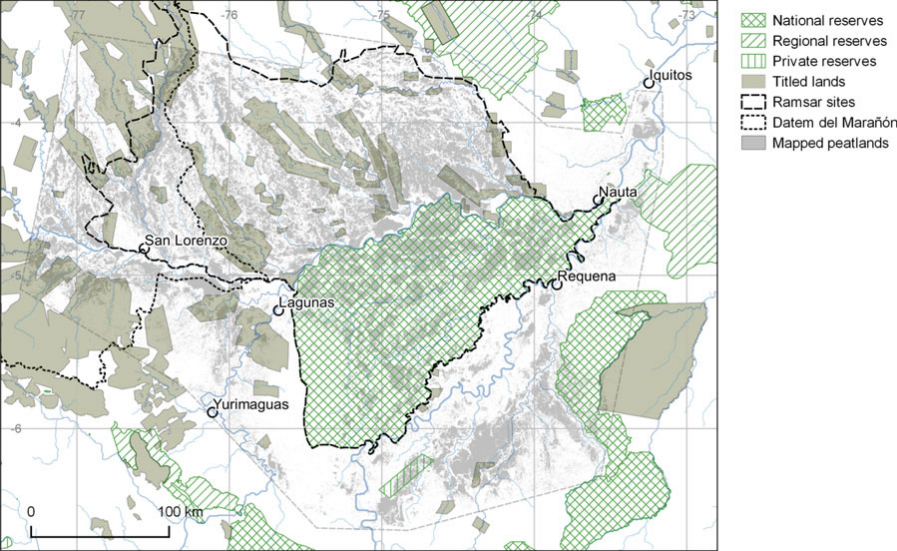
Protected areas and titled land (i.e., land owned formally by forest communities) in the Pastaza‐Marañón Foreland Basin, Peru (see Supporting Information for sources).

Three key trends in conservation in the PMFB suggest pathways for protecting intact tropical peatlands in general: growing interest in conservation of carbon‐rich areas as a means of climate‐change mitigation; involvement of local communities in advocating for forest protection; and continuing improvements in legal protection of areas for conservation (Fig. 5). All 3 should be pursued to preserve intact peatlands and carbon stores while facilitating sustainable development of forest communities.

### Carbon‐Based Conservation

Payments for carbon conservation (e.g., via REDD+ [Reducing Emissions from Deforestation and Forest Degradation] and the Green Climate Fund) present perhaps the most obvious route to protecting peatlands from avoidable human impacts because the high carbon density of peatlands makes for a favorable return from carbon conservation investments. The UN's REDD+ involves monitoring the 5 carbon pools identified by the Intergovernmental Panel on Climate Change (Penman et al. 2003), including belowground biomass and soil organic matter, which means peat carbon stocks should be explicitly taken into account. In the PMFB, 69% of all peatland and 67% of the exceptionally carbon‐dense peatland pole forests are currently unprotected. Pole forests, especially those close to planned infrastructure developments, present an obvious target for carbon conservation projects.

There are 2 key challenges to protecting peatlands through carbon conservation schemes. First, better spatial models of peat distribution, based on an improved understanding of peatland expression in remote‐sensing data, are needed to support carbon conservation schemes because BGC is presently difficult to map and monitor by remote sensing. Second, the current focus on AGC in carbon conservation amplifies the threat to peatland carbon stocks because peatland forests in Amazonia support much less AGC than terra firme forest. For example, in a detailed lidar‐ and Landsat‐based AGC map of Peru (Asner et al. 2014), the PMFB is conspicuous as an island of apparently low AGC within a sea of high‐AGC terra firme forest. The distribution, size, and vulnerability of BGC stocks must be made equally visible to stakeholders and policy makers to avoid the risk that agricultural expansion could be diverted from terra firme forest into peatlands.

### Local Communities

Key to the conservation of peatlands in the PMFB, and other intact tropical peatlands, are the people who live in these regions. There are many examples of efforts in the PMFB by communities, often assisted by governmental and nongovernmental agencies, to develop sustainable resource‐management strategies (e.g., for fisheries and *M. flexuosa* fruit harvesting) that are consistent with peatland conservation in the wider wetland ecosystem (e.g., Janovec et al. 2013). Elsewhere in Amazonia, conferring land tenure to indigenous communities, as distinct from more recent immigrants, has been argued to be particularly effective in protecting forests against the expanding agricultural frontier (Oliveira et al. 2007). Numerous ethnic groups, including the Candoshi, Awajún, Achuar, Shapra, Urarina, and some Wampis and Shuar, live north of the Rio Marañón, where peatland carbon stocks are highest. Active programs exist with the aim of conferring land title to indigenous communities, but the process is highly bureaucratic, progress is slow (AIDESEP 2015), and it does not always adequately reflect the often extensive hinterlands exploited by communities (Gilmore et al. 2013). Titled lands (i.e., land owned formally by forest communities) currently encompass just 250,000 ha (7%) of the total PMFB peatland area and 10,000 ha (3.6%) of pole forest (Table 1). Improvements to land‐titling processes could therefore deliver both fairer outcomes for marginalized communities and an expansion of the area protected, particularly given that there is, for the time being, less competition from commercial interests for rights to exploit peatlands relative to terra firme forests. Better documentation of the extensive range of ecosystem services and other benefits provided by peatlands to forest communities would also help make the case for including large, hydrologically coherent areas of peatlands in land‐titling agreements.

**Table 1:** Area of peatland and mass of carbon (percentages of the total) stored within the Pastaza‐Marañón Foreland Basin, as modeled by Draper et al. 2014, and within the different classes of land protection in the basin

Peatland type	National, regional, or private reserves	Titled land^a^	Other
All			
area, in millions of ha (%)	0.84 (23.9)	0.25 (7.0)	2.44 (69.1)
carbon mass, Gt C (%)	0.76 (24.4)	0.21 (6.8)	2.13 (68.8)
Pole forest			
area, in millions of ha (%)	0.10 (29.0)	0.01 (3.6)	0.24 (67.4)
carbon mass, Gt C (%)	0.14 (29.0)	0.02 (3.6)	0.34 (67.4)
Palm swamp			
area, in millions of ha (%)	0.68 (24.5)	0.21 (7.7)	1.87 (67.8)
carbon mass, Gt C (%)	0.57 (24.5)	0.18 (7.7)	1.58 (67.8)
Open			
area, in millions of ha (%)	0.06 (15.0)	0.02 (5.5)	0.33 (79.5)
carbon mass, Gt C (%)	0.04 (15.0)	0.02 (5.5)	0.22 (79.5)

a Land owned formally by forest communities.

### Protected Areas

Legally implemented reserves may offer a high level of ecological protection by privileging conservation over development. The Pacaya‐Samiria National Reserve, designated in 1972 and managed by the Peruvian National Parks service covers 2.1 million ha and is the largest reserve in the PMFB. Other national and regional reserves (Tamshiyacu Tahuayo, Matsés, Nanay) span another 340,000 ha; together, they cover 840,000 ha (23%) of the peatlands (Table 1). Even in these reserves, some oil extraction and agriculture is permitted (Dourojeanni 2015), but overall they have effectively constrained potentially damaging development. New protected areas have been designated by the Peruvian government, such as the 1.3‐million ha Parque Nacional Sierra del Divisor, southeast of PMFB (MINAM 2015a), and the 391,000‐ha Área de Conservación Regional Maijuna Kichwa, which is comanaged by the national government and Maijuna and Kichwa communities (MINAM 2015b). Private individuals and nongovernmental organizations (NGOs) have also been effective in protecting tracts of land. Several, small, privately owned reserves (up to 100 ha [Fig. 5]) exist in and around the PMFB but are too small to protect whole watersheds and maintain peatland integrity. An additional model of conservation is the Yanayacu‐Maquia Concession, a renewable 40‐year conservation concession covering 38,700 ha of the southern PMFB, granted by the Peruvian government in 2006 to an NGO, Conservación Amazónica. Thus, several governance models can and should be used as soon as possible to increase the area protected within the PMFB and elsewhere before commercial interests expand into these regions and make protection politically infeasible.

The Pacaya‐Samiria National Reserve is listed as a wetland of international importance under the terms of the Ramsar Convention (Ramsar 2016) and was so designated (along with the adjacent Abanico del Pastaza Wetland) in 2002 on the basis of evidence assembled by the World Wildlife Fund. These Ramsar sites together encompass 63.7% (2.24 million ha) of the modeled peatland area. Ramsar designation does not afford formal legal protection, but it does require an ecological inventory and development of a management plan, both of which are significant challenges (given the scale of the sites) that have yet to be met (Ramsar 2016). Ramsar designation does, however, provide a basis from which to develop further layers of protection with the assistance of an international community of experts in wetland management. Expansion of the Ramsar designation to encompass the entire PMFB and seeking Ramsar designation for other intact peatlands across the tropics could be useful steps in facilitating formal legal protection.

## Conservation Implications for Intact Tropical Peatlands Globally

Although the sociopolitical context differs from one region to another, the general trends facing the conservation of intact tropical peatlands are similar: thousands of kilometers of road and railroad are being built or upgraded (Weng et al. 2013; Laurance et al. 2015); numerous hydropower projects are being implemented (Winemiller et al. 2016); oil‐palm plantations are expanding (Sayer et al. 2012); and extraction of various mineral resources, not only oil and gas, is also widespread (Weng et al. 2013). Further research to determine the vulnerability of tropical peatlands to future climate change is badly needed. However, it seems that across key regions with intact lowland tropical peatlands (including western Amazonia and western and central Africa) 21^st^ century climatic warming will be offset by increased rainfall, suggesting that the carbon storage function of peatlands here may be preserved.

Based on our analysis of the opportunities for conserving PMFB peatlands, we came to 3 conclusions which may apply widely across the tropics and be tailored to local circumstances. First, integrating carbon‐based initiatives and indigenous interests with traditional biodiversity conservation may provide a compelling basis for formal legal protection of tropical peatlands. A promising example of this integrated approach in the PMFB is the very first project to be funded by the Green Climate Fund (http://www.greenclimate.fund). An investment of $10.1 M in Datem del Marañón Province, which intersects the western edge of the PMFB (Figure 5), will promote and develop sustainable biobusinesses run by indigenous communities living along the Pastaza and Morona Rivers. The project aims to increase the incomes of these communities through sustainable harvesting of forest products, while protecting peatland carbon stocks. Similar projects could potentially be implemented across many tropical peatlands and would benefit from monitoring and evaluation of ongoing peatland conservation projects in different social and ecological contexts.

Second, established routes for conservation such as land titling for indigenous communities and reserve designation remain highly relevant, but there is room for more extensive and effective implementation. With the involvement of indigenous communities, several new regional conservation areas, such as the Área de Conservación Regional Maijuna Kichwa in Loreto, have been declared in recent years in northern Peru. Our carbon mapping and analysis of threats suggest that similar schemes should be applied, as a priority, to the 240,000 ha of currently unprotected but especially carbon‐dense and sensitive domed pole forest peatlands in the PMFB. More widely, peatland ecosystems, in parts of the Congo Basin, for example, are not yet contested or subjected to land grabs by corporations or states yet are still valuable to the people who use them. Recognition of the potential biodiversity and carbon‐based arguments for conservation of peatlands, coupled with the extra funding opportunities that the presence of peat brings, should enable these forms of protection to be applied while the land is still perceived to be of little conventional economic value.

Third, scientific research can help address the challenges involved in developing carbon‐conservation projects. In particular, our experience in the PMFB shows that robust and detailed modeling of the distribution of BGC is a prerequisite for strategic planning for carbon conservation. Remote‐sensing approaches to peat‐distribution modeling are increasingly well established, although field work is still vital for validating the results. Carbon‐density measurements, biodiversity mapping, and long‐term monitoring of water balance and carbon fluxes should be a research priority, particularly in Africa and New Guinea, where few reliable data are available.

The unfortunate history of Indonesian and Malaysian peat swamp forests, which seems destined to result in their near‐total loss, is a stark indication of one possible future for currently intact tropical peatlands. Preemptive conservation action founded on peat‐distribution modeling and field mapping, a robust understanding of the consequences of peatland drainage and land‐use conversion, and analysis of local threats and opportunities should help avoid further unnecessary ecological losses and enhanced greenhouse gas emissions from the remaining intact peatlands across the tropics.

## Supporting information

Data sources used in figures and tables (Appendix S1) and a Spanish translation of the article (Appendix S2) are available online. The authors are solely responsible for the content and functionality of these materials. Queries (other than absence of the material) should be directed to the corresponding author.

Supporting Information
