# Supplementary material for: Threats to intact tropical peatlands and opportunities for their conservation
Source: Conserv Biol. 2017 Jul 10;31(6):1283–92. doi: 10.1111/cobi.12925 (PMC6849624; doi:10.1111/cobi.12925)
Supplement: Supplementary file 1 — Data sources used in figures and tables (Appendix S1) and a Spanish translation of the article (Appendix S2) are available online. The authors are solely responsible for the content and functionality of these materials. Queries (other than absence of the material) should be directed to the corresponding author. [file COBI-31-1283-s001.docx]

# Data sources used in maps and tables

**Figure 1. Mapped peatlands in the Amazon basin.**

**Pastaza-Marañón Foreland Basin:** Lähteenoja et al. 2009a, b, 2012; Lähteenoja and Page 2012; Draper et al. 2014

**Peruvian Amazon River floodplain:** Lähteenoja et al. 2009a

**Madre de Díos:** Householder et al. 2012

**Rio Negro basin:** Lähteenoja et al. 2013

**Figure 2. Vegetation distribution and inferred below-ground carbon storage in the peatlands of the Pastaza-Marañón Foreland Basin.**

**Vegetation distribution, mapping boundary:** Draper et al. 2014

**Watercourses:** hydrosheds.cr.usgs.gov.

**Towns:** maps.google.com

**Figure 3. Potential threats to carbon storage and biodiversity in the peatlands of the Pastaza-Marañón Foreland Basin.**

**Oil palm locations:** EIA (2015); USAid (2015); some proposed sites (Qda. Curaca, Caserio Huambe) are mapped in <http://www.biofuelobservatory.org/Documentos/Submission-to-the-USTR/3ra-Comunicacion/Informe-017-2013-SERNANP-RNAM-JRR-Sobrevuelo-02-agosto.pdf>, dated 2/8/13; see also maaproject.org especially Finer M, Novoa S (2015) Deforestación por Palma Aceitera en la Amazonía Peruana (Parte 2: Shanusi). MAAP: Image #16 <http://maaproject.org/2015/10/imagen16-shanusi/>, which suggests that the area of deforestation is slightly larger than that mapped in our Figure 3. The Quebrada Curaca lots are also shown in an article in El Comercio, 17/3/13, based on Finer/MAAP: <http://www.biofuelobservatory.org/Documentos/Submission-to-the-USTR/4ta-Comunicacion/II.-Aprobacion-EIA-Manati/II.6-Publicaciones-El-Comercio.pdf>The Q

**Oil lots:** Perupetro (November 2015) <http://www.perupetro.com.pe/wps/wcm/connect/perupetro/site/Informacion%20Relevante/Mapa%20de%20Lotes/Mapa%20de%20Lotes>, accessed 16/12/15, dataset dated 28/11/15.

**Productive oil wells:** Perupetro (November 2015) <http://www.perupetro.com.pe/wps/wcm/connect/c5d86c7f-9e65-43e1-b314-302c8d156e4b/1_Nov_Mapa+de+Pozos+Exploratorios.pdf?MOD=AJPERES>, accessed 24/1/16.

**Oil pipelines:** Dourojeanni (2013), verified against major pipeline routes published by Perupetro (December 2015). <http://www.perupetro.com.pe/wps/wcm/connect/3b5b9fce-a1aa-4482-87ef-7b541f94e817/CNT%2BDic%2B2015.pdf?MOD=AJPERES&Mapa%20Lotes%202015-12>, accessed 24/1/16, source map dated December 2015. The Dourojeanni map includes two short sections of pipeline associated with the Corrientes wells in Lot 8 which are not shown in the PeruPetro map.

**Mazán dam location:** [www.dams-info.org](http://www.dams-info.org) accessed 16/12/15, and Finer 2012.

**Proposed Yurimaguas-Iquitos railroad:** Redrawn from Congreso de la Republica del Perú (2013) *Informe de actividades de representación Oficio No. 043-2013-2014/VIR-CR and Oficio Circular No 011-2013-DP-OM/CR*, dated 9/9/13, (<http://www2.congreso.gob.pe/Sicr/MesaDirectiva/sipfr2011.nsf/D71940A0FEAD24A505257BE8006B51CF/$FILE/IslaRojas_AGO2013.pdf>, accessed 22/1/16), cross-checked against La Región (2012) *Una cosa es que no se pueda hacer trabajos en la selva por lluvias y otras no se pueda proyectar un tren* <http://diariolaregion.com/web/una-cosa-es-que-no-se-pueda-hacer-trabajos-en-la-selva-por-lluvias-y-otras-que-no-se-pueda-proyectar-un-tren/>, accessed 22/1/16) and Proyecto Loreto Sostenible (2013) *Loreto 2013: Análisis del estado de los sectores principales del desarrollo.* (<https://foroloreto.files.wordpress.com/2013/11/synthesis-report_transportacion.pdf>, accessed 22/1/16)

**Proposed Moyobamba-Iquitos electricity transmission line:** Redrawn from Lineas de Transmision Peruanas (2015) Estudio de Impact Ambiental: Línea de Transmisión 220kV Moyobamba – Iquitos y Subestaciones Asociadas. <http://www.minem.gob.pe/minem/archivos/file/DGGAE/DGGAE/ARCHIVOS/estudios/EIAS%20-%20electricidad/EIA/EIA-MOYOBAMBA-IQUITOS-2015/TOMO%20I_FOL.pdf?ldnhfbclwsksksbt?ydgwzpceiqqezfbz> (accessed 22/1/16).

**Road projects:** redrawn from <http://www.mtc.gob.pe/estadisticas/mapas_departamentales/16_LORETO-Intervenciones%20RVN_Telecom.pdf>, confirmed against <http://gestion.pe/economia/mtc-invertira-s-2940-millones-obras-infraestructura-region-loreto-2121182> (news article dated 21/1/15, accessed 16/12/15) which confirms that the Mazan and Jenaro Herrera-Colonia Angermos road projects have been allocated funding, and against Dourojeanni, M. (2013) Loreto Sostenible al 2021. Derecho Ambiente y Recursos Naturales, Lima, which indicates additional projects.

Other data as in Figure 2.

**Figure 4. Protected areas in the Pastaza-Marañón Foreland Basin.**

**Protected areas:** WDPA (2015) and SERNANP (2015); boundary of the Yanayacu reserve redrawn from INRENA (no date) Mapa de Ubicacion: Concesion de Conservación, Lotty del Carmen Morey Amasifuen, Contrato No 16-Con/Con-J-001-006 (available at <http://www.biodiversite-amazonienne.com/IMG/jpg_carte_officiel_YM_BD.jpg> accessed 24/1/16).

**Boundary of the Abanico de Pastaza Ramsar site:** Redrawn from INRENA (2002) Propuesta Ramsar Abanico del Pastaza (<https://rsis.ramsar.org/RISapp/files/689/pictures/PE1174map.pdf>, accessed 24/1/16).

**Titled lands:** RAISG 2012, “Amazon 2012 Protected Areas Indigenous Territories.” <http://raisg.socioambiental.org/system/files/AMAZON2012_english.pdf>, data source <http://gisserver.socioambiental.org:6080/arcgis/services/raisg/raisg_tis/MapServer/WMSServer?request=GetCapabilities&service=WMS> (accessed 16/12/15).

Other data as in Figure 2.

**Additional references**

Dourojeanni, M. (2013) *Loreto Sostenible al 2021*. Derecho Ambiente y Recursos Naturales, Lima, 165 pp.
