# Supplementary material for: Threats to intact tropical peatlands and opportunities for their conservation
Source: Conserv Biol. 2017 Jul 10;31(6):1283–92. doi: 10.1111/cobi.12925 (PMC6849624; doi:10.1111/cobi.12925)
Supplement: Supplementary file 2 — Supporting Information [file COBI-31-1283-s002.docx]

# Amenazas a las turberas tropicales intactas y oportunidades para su conservación

## Resumen

Extensas e intactas áreas de turberas tropicales están severamente amenazadas a escala mundial, debido a la expansión de la agricultura comercial y a otras formas de desarrollo económico. Al nivel del paisaje, la conservación de las turberas sin intervencion a su hidrología es de importancia internacional para asegurar la conservación de su biodiversidad tan característica y de los servicios ambientales que ofrecen, y así mantener su resiliencia ante futuros cambios ambientales. Exploramos las amenazas y las oportunidades para conservar las turberas tropicales intactas restantes. Nuestro enfoque por lo tanto excluye las turberas de Indonesia y Malasia, donde la deforestación de forma extensiva y el drenado para el establecimiento de plantaciones hace que la conservación en estas regiones pueda proteger solo pequeños fragmentos del ecosistema original. En este estudio, nos enfocamos en las cuencas del Pastaza-Marañón (PMFB, siglas en inglés) en Perú, que comprende una de las turberas tropicales intactas más extensas del mundo y es representativa de la vulnerabilidad que las amenaza. El mantenimiento de las condiciones hidrológicas críticas para el almacenamiento de carbono y la función ambiental de las turberas está, en la PMFB, amenazada principalmente por la expansión de la agricultura comercial y las nuevas vías de transporte que facilitan el acceso a las áreas remotas. En la PMFB y en otros lugares aún hay oportunidades para desarrollar prácticas alternativas y sostenibles del uso del suelo. Aunque algunas de las turberas en la PMFB caen dentro de áreas naturales protegidas, esta protección no incluye las áreas con mayor densidad de carbono (varillal hidromórfico). Nuevas herramientas para la conservación basada en el carbono (p.e. REDD+, Fondo Verde para el Clima), el desarrollo de mercados para los productos de las turberas, la transferencia de títulos de propiedad de la tierra a las comunidades locales, y la expansión de las áreas protegidas ofrecen alternativas para una mayor protección de las turberas tropicales intactas en la Amazonía y en otros lugares, como las turberas de Nueva Guinea y África Central que permanecen, por el momento, alejadas de la frontera de desarrollo comercial.

**Palabras claves:** Amazonía, carbono, turbera, Perú, turba, trópicos

## Introducción

Los incendios catastróficos a finales del 2015 en Indonesia (Chisholm et al., 2016) representan el último episodio de la destrucción y degradación de algunos de los ecosistemas de turberas más extensos de los trópicos. Estos incendios resaltan la falta de protección de estos sistemas densos en carbono, que cuando se combinan con los efectos del cambio en el uso de la tierra, el drenaje y las sequías episódicas de El Niño, conducen no solo a la pérdida de hábitats, sino también a la emisión del carbono almacenado debajo del suelo (BGC, siglas en inglés – ‘Below-Ground Carbon’) hacia la atmósfera. Afortunadamente, los ecosistemas de turberas tropicales en la Amazonía, África y Nueva Guinea son también extensos y generalmente explotados con menor intensidad. Muchos de estos ecosistemas pueden ser descritos como intactos a nivel de paisaje; es decir, su hidrología no está afectada por la actividad humana y la cobertura vegetal no está fragmentada o sustancialmente degradada. Sin embargo, su importancia se articula débilmente dentro de las agendas de conservación existentes, principalmente porque las turberas no están bien descritas y cartografiadas, y frecuentemente no son reconocidas por las agencias e instituciones locales. Aquí discutimos los servicios proporcionados por las extensas e intactas turberas tropicales, las amenazas que enfrentan y las oportunidades que existen para conservarlas.

La mayor parte de nuestros ejemplos provienen de las cuencas del Pastaza-Marañón (PMFB, siglas en inglés) ubicadas en la Amazonía baja peruana (Figura 1. Estas cuencas presentan gran extensión y alta densidad de carbono en sus turberas, desconocidas hasta hace muy poco y por lo tanto han generado investigación científica y la reevaluación de las estrategias de conservación con el potencial de ser aplicadas en otros lugares. La existencia de depósitos de turba en esta parte de la cuenca amazónica fue documentada a principios del 2000 (Schulman et al., 1999, Ruokolainen et al., 2001, Freitas et al., 2006) y el primer estudio sistemático de su espesor y extensión fue publicado por Lähteenoja et al. (2009a, b). Hasta 7.5 m de turba fue acumulada en la PMFB durante los últimos 8900 años (Lähteenoja et al., 2012) en un área que cubre cerca de 3.5 millones de hectáreas (Draper et al., 2014). Las estimaciones más actuales de la distribución de la turba a nivel de los trópicos (Page et al., 2011), incluyendo los nuevos descubrimientos en la Cuvette Centrale de la Cuenca del Congo (Dargie et al., 2002), sugieren que las ca. 3 Gt de carbono almacenados en la PMFB (Draper et al., 2014) representan casi el 2.7 % del total de las reservas de carbono en las turberas tropicales. El almacenamiento de carbono en las turberas está muy relacionado con la saturación del agua en el suelo, lo que favorece las condiciones anaeróbicas que limitan la descomposición de la materia orgánica. El drenaje que se requiere para convertir las turberas a otros usos, como a plantaciones de palma aceitera, típicamente conducen a la rápida descomposición aeróbica de la turba, la emisión de carbono a la atmósfera, y las pérdidas del carbono arriba del suelo (AGC, siglas en Inglés – ‘Above-Ground Carbon’) y de la biodiversidad. Por lo tanto, las turberas son vulnerables a las perturbaciones y degradaciones humanas, de manera cualitativamente diferente a otros ecosistemas forestales, y las emisiones de carbono por área pueden resultar desproporcionadamente mayores.

Al igual que sus contrapartes en el sudeste asiático, las turberas del PMFB se forman en diversos tipos de vegetación (Figuras 2 y 3, Draper et al., 2014). El tipo de vegetación más extensa es el pantano de palmeras o aguajal, caracterizado por la abundante presencia de la palmera *Mauritia flexuosa*, y cubre 2.8 Mha. El varillal hidromórfico se caracteriza por la presencia de árboles de baja estatura y delgados, y cubre 350,000 ha; está restringido a zonas donde existen turberas elevadas en forma de domo, pobres en nutrientes y alimentadas por la lluvia, y tiene una estructura similar a los bosques de turbera ombrotrófica del sudeste asiático (Anderson 1983). El varillal hidromórfico es el ecosistema más denso en carbono que se conoce en la Amazonía, con 1391 ± 710 Mg C/ha almacenado en su mayoría debajo del suelo (Draper et al., 2014). El tercer tipo de vegetación es el pantano abierto (410,000 ha), dominado por comunidades herbáceas que aún no han sido descritas en detalle.

En comparación con los bosques de tierra firme sumamente diversos en la región, los tres tipos de vegetación de turbera tienen una baja diversidad (α). Sin embargo, se diferencian a nivel florístico porque son dominados por un pequeño número de especies especialistas, aunque también presentan especies comunes a los bosques de tierra firme (Draper 2015). Esta diferenciación en su composición probablemente refleja la fisiología exigente del hábitat (anoxia, anegamiento y, en el caso de los domos de turba, deficiencia de nutrientes [Rydin & Jeglum 2013]) y los efectos de la perturbación a escalas centenarias a milenarias a través de la migración de los canales fluviales (Roucoux et al. 2013).

La densidad poblacional humana en la PMFB es baja (2.4 personas/km en Loreto [INEI 2015]), pero sus ecosistemas presentan intervención y no son vírgenes. Los pantanos de palmeras son reconocidos por las comunidades bosquesinas como hábitats ricos en recursos, y son utilizados para cazar y recolectar productos vegetales. Más de 50 especies de plantas son útiles para la construcción, alimentación, medicina y motivos ceremoniales, y varios productos forestales son parte de la economía (p.e., los frutos de *M. flexuosa* orquídea de vainilla) (Householder et al. 2010; Gilmore et al. 2013).

Las turberas en el PMFB son también hábitats importantes para los animales. En los pantanos de palmeras, *M. flexuosa* produce frutos abundantes y nutritivos que sustenta a una fauna diversa y densa, incluyendo monos, tapires (*Tapirus terrestris*), pecaríes (*Tayassu pecari*), agutíes (*Agouti paca*), guacamayos (p.e. *Ara* sp.), tortugas y peces (Gilmore et al., 2013). Además, los varillales hidromórficos albergan varias especies de aves amenazadas y en peligro de extinción, que han sido sólo reportadas en bosques cercanos de arena blanca igualmente pobres en nutrientes (Lähteenoja et al., 2009b). Estos ecosistemas presentan flora y fauna distintivas y características, de valor similar a la biodiversidad de las turberas del sudeste asiático (Wich et al., 2016).

## Amenazas potenciales a los ecosistemas de turberas tropicales

### Vías de transporte

El desarrollo de vías de transporte en los bosques tropicales típicamente acelera la degradación de los bosques y su deforestación (Laurance et al., 2009). El impacto es particularmente severo cuando estas actividades se realizan por primera vez en bosques no intervenidos (Laurance et al., 2015), un escenario típico de las zonas de turberas tropicales intactas. Por ejemplo, actualmente en la PMFB hay pocas carreteras y ningún ferrocarril, y la mayoría de personas y mercancías viajan por los ríos (Figura 4). Sin embargo, se plantean varios proyectos de infraestructura, incluyendo las primeras carreteras pavimentadas para conectar la PMFB con el resto del Perú, Brasil y Colombia; el mejoramiento de la navegabilidad y de las instalaciones portuarias a lo largo de las vías fluviales importantes (hidrovías); y la implementación de una línea de transmisión de electricidad y su vía de servicio entre Moyobamba y la capital regional, Iquitos (Dourojeanni 2016; La Región 2016). Algunas de estas rutas planificadas pasan directamente por las regiones donde se concentran los varillales hidromórficos (Figura 4).

En la PMFB, esta nueva infraestructura puede tener un efecto transformador al reducir los costos de transporte y alentar las inversiones de la agricultura comercial. El mejoramiento del acceso a los mercados para los pueblos y las comunidades, previamente aisladas, también promueve la inmigración y la expansión agrícola de pequeños agricultores. Por ejemplo, en las regiones mejor conectadas de la Amazonía, la inmigración es significativa (p.e., 6% al año en Madre de Dios, en el sur del Perú), y los pequeños agricultores han aprovechado los mercados existentes y han utilizado trabajadores inmigrantes para aumentar las áreas de cultivo (Ichikawa et al. 2014). La construcción de carreteras o vías de ferrocarril por las turberas podría alterar las redes de drenaje y la conectividad hidrológica, aumentando así, el potencial de las consecuencias negativas para los niveles de la capa freática, las vías de drenaje y los períodos de inundación (Barry et al., 1992), que son fundamentales para mantener la integridad de las turberas y su función de almacenamiento del carbono.

### Agricultura

La expansión de plantaciones de palma aceitera fue el motor principal de la deforestación de las turberas tropicales en Indonesia y Malasia, y este modelo de desarrollo económico se está expandiendo a áreas boscosas en otras regiones que no han sido intervenidas. Por ejemplo, las plantaciones comerciales se están expandiendo rápidamente en la Amazonía occidental (Figura 4, Gutiérrez-Vélez et al., 2011), ya que la demanda por el aceite de palma está creciendo y las nuevas variedades aumentan la rentabilidad del cultivo (Villela y otros, 2014). En el departamento de Ucayali en el Perú, >9400 hectáreas de bosques primarios de *tierra firme* fueron deforestados para las plantaciones de palma aceitera desde el 2011 (Erickson-Davis 2015). Al lado este del PMFB, 2126 ha de bosque primario fueron deforestadas para el cultivo del cacao y palma aceitera entre mayo de 2013 y agosto de 2014 por la United Cacao; la compañía posee otras tierras cercanas y existe la posibilidad de un incremento aún mas grande de deforestación en la zona (Finer & Novoa 2015). La agricultura comercial aún no se ha expandido a las turberas de la PMFB, pero las plantaciones y los arrozales avanzan hacia los humedales y los pantanos de palmeras en otras partes del Perú, como en la región de Madre de Dios (Janovec et al. 2015) y en Colombia (Potter 2015).

La experiencia de Indonesia y Malasia demuestra de manera dramatica el impacto que produce convertir las turberas a zonas agrícolas. Típicamente, las plantaciones de palma aceitera en esta región están establecidas en domos de turbera pobres en nutrientes. El drenaje causa que la turba experimente una descomposición acelerada, compactación y oxidación (Moore et al., 2013). La capa superficial de la turba seca se incendia fácilmente y los incendios que se propagan pueden emitir cantidades globales significantes de carbono, normalmente 0.2 Gt C/año en las últimas décadas y hasta 0.7 Gt C/año durante los años afectados por El Niño como en el 1997 (Dommain et al., 2014). Hergoualc'h y Verchot (2011) estimaron de forma conservadora la pérdida de carbono en 427.2 ± 90.7 Mg C/ha durante los primeros 25 años del ciclo de rotación de la palma aceitera.

Ciertamente, mientras la expansión de las plantaciones en bosques de tierra firme sea posible, la conversión de las turberas es comparativamente poco atractiva debido a los costos de drenaje y fertilización, y la escasez de especies forestales maderables valiosas aprovechables durante la deforestación de estas zonas. Sin embargo, cualquier fortalecimiento de la protección de los bosques primarios de tierra firme, sin el mejoramiento de la protección de las turberas, aumentaría el riesgo de expansión agrícola en estas turberas (actualmente) menos viables. La gravedad del potencial impacto ambiental proporciona una sólida justificación para establecer una barrera legal contra la expansión agrícola en las turberas. En el Perú todas las propuestas de desarrollo, incluidas las plantaciones, deben someter una evaluación formal del impacto ambiental, que debería incluir la mitigación de las amenazas a las turberas siempre y cuando se reconozca la presencia del carbono debajo del suelo. Sin embargo, dicha protección jurídica no siempre es eficaz; se alega que algunas plantaciones se han establecido ilegalmente y sin una evaluación adecuada del impacto ambiental (EIA 2015). En tales casos los tribunales pueden intervenir, aunque a veces la intervención se realiza después de que el daño se produjo (EIA 2015, USAID 2015). Las plantaciones legales de palma aceitera también pueden tener un impacto ambiental considerable. Por ejemplo, existe un preocupante precedente legal, donde se utilizó la política de promoción de la producción de biocombustibles para justificar y obtener permiso para establecer plantaciones en bosques primarios del departamento de San Martín, Perú (Potter 2015), incluyendo pantanos de palmeras (D.d.C.T., observación personal).

### Actividades de los pequeños agricultores

La actividad de los pequeños agricultores representa un importante uso de la tierra dentro y alrededor de las turberas tropicales intactas. En la PMFB, el cultivo de estos agricultores se restringe a suelos aluviales limosos, pero los pantanos de palma (incluyendo los de turba) son frecuentemente visitados por pequeños propietarios para recolectar recursos silvestres y cazar. Los impactos ecológicos pueden ser considerables en sitios de acceso fácil al mercado principal de la región de Iquitos. Las formas no sustentables de cosecha del fruto de la *M. flexuosa*, que incluye la tala de árboles frutales femeninos (Gilmore et al., 2013) y la producción de carbón vegetal (Arce-Nazario 2007), son ampliamente practicadas. En Iquitos se consumen ~130 t. de frutos de *M. flexuosa* cada mes, que son el producto de ~1078 árboles (la mayoría de los cuales han sido talados para cosechar el fruto), que proveería un sustento para ~4700 personas (Delgado et al., 2007, Rojas et al., 2001). Estas actividades afectan directamente al ambiente vegetal, por ejemplo, cambiando la proporción de sexos masculino-femenino de las poblaciones de *M. flexuosa* (Horn et al., 2012) y alentando un incremento en la abundancia de árboles dicotiledóneos en lugar de los árboles de palma (Endress et al., 2013). No se sabe si la hidrología de las turberas está directamente afectada; sin embargo, en su escala actual, es poco probable que estos pequeños impactos sean un factor importante de degradación de las turberas. De todos modos, es probable que el crecimiento de las plantaciones comerciales en la región estimule la participación de los pequeños agricultores en las economías comerciales (por ejemplo, la palma aceitera), como ha ocurrido en otros lugares (Sayer et al., 2012). Por lo tanto, aunque actualmente el riesgo de una degradación general de las turberas por parte de la agricultura comercial y de pequeños agricultores parece ser bajo, es incierto si este riesgo seguirá siendo igualmente bajo en el futuro.

### Cambio climático

La amenaza principal a las turberas asociadas al cambio climático es el aumento de la duración y/o gravedad de las sequías que, al reducir la capa freática, conducirían a una rápida degradación de la turba por oxidación y el probable aumento de combustibilidad (Turetsky et al., 2015). Los registros paleo-ecológicos sugieren que la acumulación de turba ha sido sensible al cambio climático del pasado: por ejemplo, un registro de la turbera de San Jorge en la PMFB indica una pronunciada reducción en la tasa de acumulación de turba entre 650 y 1550 d.C., que parece coherente con una desecación climática (Kelly et al., 2016). Aunque las predicciones del cambio climático en el futuro para las áreas remotas de turberas tropicales vírgenes son inciertas, la trayectoria es probable que varíe entre las regiones. Por ejemplo, para la Amazonía occidental, los modelos climáticos suelen predecir una mayor precipitación y mayores niveles máximos de descarga de los ríos (Sorribas et al., 2016, Zulkafli et al., 2016) y a medida que avanza este siglo con menos sequías, y de menor severidad (por ejemplo, Marengo et al. 2012, Langerwische et al., 2013, Sánchez et al., 2015). Con aumentos de temperatura de ~2–4 °C, los efectos generales de la proporción entre precipitación y evaporación aproximadamente se anularían (Marengo et al., 2012). Por lo tanto, en términos de su balance hídrico, las turberas en la PMFB posiblamente pueden escapar de los peores efectos del cambio climático del siglo XXI, en contraste con las turberas del sudeste asiático que están fuertemente influenciadas por las sequías producidas por El Niño. Este riesgo, comparativamente bajo, de un incremento de sequías en la PMFB aumenta el valor de esta región como una reserva de carbono a largo plazo que merece protección.

### Minería, petróleo y gas

La extracción de recursos minerales y de hidrocarburos puede causar una deforestación sustancial tanto directa como indirecta. En la PMFB la exploración y extracción de hidrocarburos geológicos es muy activa (Finer et al., 2015) y cerca del 24% del área de turba en la PMFB se encuentra dentro de terrenos designados para la extracción o exploración de petróleo, que también traslapa sustancialmente a las áreas protegidas y a las tierras tituladas de las comunidades indígenas. Desde 1972, PetroPeru ha operado grandes instalaciones en tres áreas separadas (Figura 4). El resultado principal de estas actividades es el desbroce para la construcción de trochas, para la instalación de tuberías y líneas de encuesta sísmica. La mayoría de las líneas de encuesta sísmica se vuelven a cubrir de vegetación, pero algunas son mantenidas abiertas por las comunidades locales; las rutas de las tuberías son rutinariamente mantenidas accesibles para fines de mantenimiento, mejorando el acceso a áreas remotas y potencialmente afectando la hidrología de las turberas si estas rutas son pavimentadas. Los derrames de petróleo son comunes a lo largo de los oleoductos (por ejemplo, La Republica 2013), pero no hay estudios de su impacto ecológico en las turberas.

### Energía hidráulica

La hidroelectricidad es central en los planes de desarrollo de la Amazonía, y el impacto de este desarrollo en la biodiversidad acuática y terrestre de la zona es una preocupación importante en el tema de conservación (Lees et al., 2016). En la Amazonía Occidental ~150 represas principales han sido planeadas, incluyendo la presa Mazan justo bajo el Canal de Iquitos. La presa más grande prevista es la mega-presa de 4500 MW Manseriche, justo arriba de la PMFB en el Río Marañón (Finer & Jenkins 2012). Las consecuencias para las turberas que se encontrarían abajo de las nuevas presas aún no han sido estudiadas, pero los impactos relevantes podían incluir cambios en la cantidad de sedimentos suspendidos, de nutrientes y de materia orgánica transportados por los ríos y cambios en el sistema fluvial (por ejemplo, reducción del flujo máximo; Ligon et al., 1995). Estas represas tienen potencialmente la capacidad de afectar el estado nutricional y la hidrología de las turberas de las zonas inundables, y las características de drenaje de las turbas, y sobre los sistemas fluviales a través de cambios en el nivel de base de los ríos. La interacción entre el cambio climático, que puede aumentar el flujo de los ríos, y el desarrollo de esquemas de energía hidroeléctrica que actúan para amortiguar los ciclos estacionales, presenta una capa adicional de complejidad en las amenazas que enfrenta la PMFB.

### Oportunidades para la conservación de turberas tropicales intactas

Tres tendencias claves de conservación en la PMFB podrian ser aplicadas de manera general para la protección de las turberas tropicales intactas: (1) creciente interés en la conservación de hábitats ricos en carbono como un medio de mitigación del cambio climático; (2) participación de las comunidades locales en la defensa de la protección de los bosques; y (3) mejoramiento continuo de la protección legal de áreas de conservación (Figura 5). Las tres deben ser incorporadas con el fin de preservar los hábitats de turberas no intervenidas y las reservas de carbono y al mismo tiempo facilitando el desarrollo sostenible de las comunidades humanas locales.

### Conservación basada en carbono

Pagos por la no emisión de carbono (por ejemplo, a través de UN-REDD+ [Reducing Emissions from Deforestation and Forest Degradation] y el recién establecido Green Climate Fund [Fondo Verde para el Clima]) quizás representan la ruta más obvia para proteger las turberas de los impactos humanos, porque la alta densidad de carbono de las turberas permite una gananza favorable de las inversiones en la conservación del carbono. REDD+ implica el monitoreo de las cinco reservas de carbono identificadas por el IPCC (Grupo Intergubernamental de Expertos sobre el Cambio Climático) (Penman et al., 2003), incluyendo la biomasa subterránea y la materia orgánica del suelo, lo que significa que las reservas de carbono de turba deben ser tomadas en cuenta explícitamente. En la PMFB, el 69% de todas las turberas y el 67% de los varillales hidromórficos densos de carbono están desprotegidos actualmente. Los varillales hidromórficos, especialmente aquellos cerca de propuestos proyectos de infraestructuras, presentan un objetivo obvio para los proyectos de conservación de carbono.

Existen dos desafíos claves para proteger las turberas a través de los esquemas de conservación del carbono. En primer lugar, se necesitan mejores modelos de distribución de turba, basados ​​en una mejor comprensión de la expresión de las turberas en los datos de teledetección, para apoyar los esquemas de conservación de carbono ya que actualmente el BGC es difícil de mapear y monitorear mediante la teledetección. En segundo lugar, el enfoque actual en AGC en la conservación del carbono amplifica la amenaza a las reservas de carbono de las turberas porque los bosques de turberas en la Amazonia apoyan mucho menos el AGC que el bosque de tierra firme. Por ejemplo, en un detallado mapa del AGC de LiDAR y Landsat del Perú (Asner et al., 2014), la PMFB es conspicua como una isla aparentemente baja de AGC dentro de un mar de tierra firme lleno de AGC. La distribución, el tamaño y la vulnerabilidad de los yacimientos de BGC deben ser igualmente evidentes para las partes interesadas y los políticos para evitar el riesgo de que la expansión agrícola pueda ser desviada de los bosques de tierra firme hacia las turberas.

### Comunidades locales

El elemento clave para la conservación de las turberas en la PMFB, y otras turberas tropicales intactas, son las personas que viven en estas regiones. Existen muchos ejemplos de esfuerzos en la PMFB por parte de las comunidades locales, asistidas por agencias gubernamentales y otras organizaciones, para desarrollar estrategias sustentables para el manejo de recursos (por ejemplo, pesquerías y cosecha de frutas de *Maurtia flexuosa*) que sean compatibles con la conservación de las turberas dentro de un ecosistema mas amplio de los humedales (por ejemplo, Janovec et al., 2013). En otras partes de la Amazonía, se discute la concesión de la tenencia de la tierra a comunidades indígenas, a diferencia de recientes inmigrantes, como una estrategia más eficaz para la protección de bosques contra la expansión agrícola (Oliveira et al., 2007). Varios grupos étnicos, incluyendo los Candoshi, Awajún, Achuar, Shapra, Urarina y algunos Wampis/Shuar, viven al norte del Río Marañón donde las reservas de carbono de las turberas son más altas. Existen programas actuales con el fin de conferir títulos de tierra a las comunidades indígenas, pero el proceso es altamente burocrático, lento (AIDESEP 2015) y no siempre refleja adecuadamente el conocimiento y uso de las extensas zonas por parte de las comunidades (Gilmore et al., 2013). Las tierras con títulos de propiedad comprenden actualmente sólo un 250,000 ha (7%) del área total de turberas en la PMFB y 10,000 ha (3.6%) de los varillales hidromórficos (Tabla 1). Por lo tanto, mejores procesos de titulación de terrenos podrían ofrecer resultados más justos para las comunidades marginadas y para la expansión de las zonas protegidas, dado que, por el momento, existe menos competencia de parte de intereses comerciales por los derechos de explotación de las turberas en comparación con los bosques de tierra firme. Una mejor documentación de la amplia gama de servicios existentes que ofrecen los ecosistemas y de los beneficios de las turberas a las comunidades amazónicas, también ayudaría a promover la inclusión de grandes áreas hidrológicamente afines de turberas en los acuerdos de titulación de tierras.

### Áreas protegidas

Las reservas establecidas legalmente tienen potencial para ofrecer una forma de protección ecológica más fuerte al privilegiar la conservación sobre el desarrollo. La Reserva Nacional Pacaya-Samiria, designada en 1972 y gestionada por el Servicio de Parques Nacionales del Perú, SERNANP, es la reserva más grande de la PMFB con una superficie de 2.1 Mha. Otras Reservas Nacionales y Áreas de Conservación Regionales (Tamshiyacu-Tahuayo, Matsés, Nanay-Pintuyacu-Chambira) cubren otras 340,000 ha; en conjunto, las áreas de conservación abarcan 0.84 Mha (23%) de las turberas (Tabla 1). Aunque en estas áreas protegidas se permite, en muy pocas cantidades, la extracción de petróleo y el uso de tierra para la agricultura (Dourojeanni 2015), en general las Reservas Nacionales y Áreas de Conservación Regionales han demostrado eficacia en limitar el potencial desarrollo dañino en estas zonas. Nuevas áreas de protección han sido designadas por el gobierno peruano, como el Parque Nacional Sierra del Divisor de 1.3 Mha al sureste de nuestra área (MINAM 2015a), y el área de Conservación Regional Maijuna-Kichwa de 391,000 ha, que es administrado por el gobierno nacional y las comunidades Maijuna y Kichwa (MINAM 2015b). Personas individuales y las ONGs también han sido eficaces en la protección de tierras. Existen pequeñas reservas privadas de hasta 100 hectáreas (Figura 5) en la PMFB y sus alrededores, pero son demasiado pequeñas para proteger cuencas hidrográficas enteras y mantener la integridad de las turberas. Un modelo adicional de conservación es la Concesión Yanayacu-Maquia, una concesión de conservación renovable de 40 años que cubre 38,700 ha. de la PMFB meridional otorgado por el gobierno peruano en el 2006 a una persona privada que creó dos organizaciones, Biodiversité Amazonienne y Conservación Amazónica, para administrar la concesión. Por lo tanto, varios modelos de gobernanza pueden y deben ser utilizados lo más pronto posible para aumentar el área protegida dentro de la PMFB, y en otras zonas. Es necesario proteger esta área antes de que la expansión territorial por parte de intereses comerciales provoque una inviabilidad política de protección en esta región.

La Reserva Nacional Pacaya-Samiria es considerada como un humedal de importancia internacional, bajo los términos de la Convención de Ramsar (Ramsar 2016), que fue designada (junto con el adyacente Humedal del Pastaza) en el 2002, con sustento técnico del World Wildlife Fund. Los dos sitios declarados por Ramsar abarcan el 63.7% (2.24 Mha) del área de turba. La designación de Ramsar no constituye una protección jurídica formal, pero requiere un inventario ecológico y el desarrollo de un plan de gestión que son desafíos importantes (dado el gran tamaño de los sitios) que todavía no se han cumplido (Ramsar, 2016). Sin embargo, la designación de Ramsar proporciona una base para desarrollar otros niveles de protección con la asistencia de una comunidad internacional de expertos en manejo de humedales. La ampliación del área designada por Ramsar para que se incluya toda la PMFB, y otras turberas intactas en los trópicos bajo está protección, podría ser un paso útil para que se facilite una protección jurídica más formal.

## Consecuencias para la conservación de las turberas tropicales vírgenes en todo el mundo

Mientras que el contexto sociopolítico difiere de una región a otra, los retos que enfrentan las turberas tropicales intactas son similares: se están construyendo o mejorando miles de kilómetros de carreteras y ferrocarriles (Weng et al., 2013, Laurance et al 2015), se están implementando numerosos proyectos hidroeléctricos (Winemiller et al 2016), las plantaciones de palma aceitera se están expandiendo (Sayer et al., 2012), y la extracción de diversos recursos minerales, no sólo petróleo y gas, se está incrementando. Se necesita de manera urgente más investigación para determinar la vulnerabilidad de las turberas tropicales dentro del futuro cambio climático. Sin embargo, parece que en turberas tropicales no intervenidas en regiones claves (incluyendo la Amazonía occidental y África occidental/central) el calentamiento climático del siglo XXI será compensado por el aumento de las precipitaciones, lo que sugiere que la función de almacenamiento de carbono de las turberas en estas regiones podría ser preservada.

Nuestro análisis de las oportunidades para la conservación de las turberas en la PMFB, nos lleva a tres conclusiones que pueden aplicarse ampliamente en los trópicos, adaptadas a las circunstancias locales. En primer lugar, la integración de las iniciativas basadas sobre el carbono y los intereses indígenas junto a una conservación tradicional hacia la biodiversidad puede proporcionar una base decisiva para la protección jurídica de las turberas tropicales. Un ejemplo prometedor de este enfoque integrado en la PMFB es el primer proyecto financiado por el Green Climate Fund (http://www.greenclimate.fund). Una inversión de $10.1 M. en la Provincia de Datem del Marañón, que traslapa el borde occidental de la PMFB (Figura 5), ​​promoverá y desarrollará "bio-negocios" sostenibles dirigidos por comunidades indígenas que viven a lo largo de los ríos Pastaza y Morona. El proyecto tiene como objetivo aumentar los ingresos de estas comunidades mediante la recolección sostenible de productos forestales, y al mismo tiempo proteger las reservas de carbono de las turberas. Proyectos símiles podrían ser implementados en muchas turberas tropicales y se beneficiarían de un monitoreo y evaluación de proyectos actuales de conservación de turberas en diferentes contextos sociales y ecológicos.

En segundo lugar, los mecanismos establecidos para la conservación como la titulación de tierras para las comunidades indígenas y la designación de áreas protegidas, siguen siendo muy relevantes, pero hay oportunidades para hacer una implementación más amplia y efectiva. Con la participación de las comunidades indígenas, nuevas áreas de conservación regional han sido declaradas, como el Área de Conservación Regional Maijuna-Kichwa en Loreto, en los últimos años en el norte del Perú. Nuestra mapeo de carbono y análisis de las amenazas sugiere que planes similares deberían ser aplicados, con prioridad, a las ˜240,000 ha. de turberas actualmente sin protección, especialmente las varillales hidromórficos, sensibles y densas en carbono, en la PMFB. En sentido más amplio, los ecosistemas de turberas (por ejemplo, en algunas partes de la cuenca del Congo) aún no han sido reclamados ni apropiados por parte de corporaciones o estados, pero siguen siendo valiosos para las personas que los utilizan. Reconociendo el valor para la conservación de la biodiversidad y del carbono, junto con las oportunidades adicionales de financiación que la presencia de turba atrae, debería permitir la aplicación de estas figuras de protección en turberas en casos donde la tierra sea percibida como de poco valor económico.

En tercer lugar, la investigación científica puede ayudar a abordar los retos que implica el desarrollo de proyectos de conservación de carbono. En particular, nuestra experiencia en la PMFB muestra que el modelo detallado y robusto de la distribución de BGC es un requisito previo para la planificación estratégica de la conservación del carbono. La teledetección del modelo de distribución de turba esta cada vez mehora elaborada. El trabajo de campo sigue siendo vital para validar los modelos de distribución de turba, los cálculos de la densidad de carbono, la cartografía de la biodiversidad, el monitoreo a largo plazo del balance hídrico y los flujos de carbono. El trabajo de campo debería ser una prioridad en la investigación, particularmente en África y Nueva Guinea donde, al presente, los datos disponibles son poco fiables.

La desafortunada historia de los bosques de pantanos de turba de Indonesia y Malasia, que parecen ser destinados a una pérdida casi total de su extensión, es una clara indicación de el posible futuro para las turberas tropicales todavía intactas. Una acción de conservación preventiva basada en el modelo de distribución de la turba, la cartografía de campo, una sólida comprensión de las consecuencias del drenaje de turberas, la conversión del uso de la tierra, y el análisis de las amenazas y oportunidades locales deberían ayudar a evitar pérdidas ecológicas innecesarias y emisiones de gases de efecto invernadero, en las restantes turberas no intervenidas en los trópicos.

## Bibliografía

Por favor vea el artículo publicado

## Leyendas de las figuras y tabla

**Tabla 1.** Área de turberas y masa de carbono (con porcentajes del total) almacenados en la PMFB, según el modelo de Draper et al. (2014), en diferentes clases de uso de la tierra.

|  | | **Áreas de conservación nacionales, regionales o privadas** | **Tierra titulada** | **Otro** |  |
| --- | --- | --- | --- | --- | --- |
|  |  |  |  |  |  |
|  |  |  |  |  |  |
|  |  |  |  |  |  |
| Todas las turbas | Área de turba (Mha) | 0.84 (23.9%) | 0.25 (7.0%) | 2.44 (69.1%) |  |
|  |  |  |  |  |  |
|  |  |  |  |  |  |
|  | Masa de carbono de turba (Gt C) | 0.76 (24.4%) | 0.21 (6.8%) | 2.13 (68.8%) |  |
|  |  |  |  |  |  |
|  |  |  |  |  |  |
| Varillales hidromórficos | Área de turba (Mha) | 0.10 (29.0%) | 0.01 (3.6%) | 0.24 (67.4%) |  |
|  |  |  |  |  |  |
|  |  |  |  |  |  |
|  | Masa de carbono de turba (Gt C) | 0.14 (29.0%) | 0.02 (3.6%) | 0.34 (67.4%) |  |
|  |  |  |  |  |  |
|  |  |  |  |  |  |
| Pantano de Palma | Área de turba (Mha) | 0.68 (24.5%) | 0.21 (7.7%) | 1.87 (67.8%) |  |
|  |  |  |  |  |  |
|  |  |  |  |  |  |
|  | Masa de carbono de turba (Gt C) | 0.57 (24.5%) | 0.18 (7.7%) | 1.58 (67.8%) |  |
|  |  |  |  |  |  |
|  |  |  |  |  |  |
| Turberas abiertas | Área de turba (Mha) | 0.06 (15.0%) | 0.02 (5.5%) | 0.33 (79.5%) |  |
|  |  |  |  |  |  |
|  |  |  |  |  |  |
|  | Masa de carbono de turba (Gt C) | 0.04 (15.0%) | 0.02 (5.5%) | 0.22 (79.5%) |  |
|  |  |  |  |  |  |
|  |  |  |  |  |  |

## Figuras

**Figura 1.** Mapa de ubicación de la cuenca amazonica (línea punteada), los ríos principales (línea continua) y las áreas de turberas (sombreadas): 1) Pastaza-Marañón Foreland Basin. 2) la planicie del río Amazonas y sus afluentes. 3) Madre de Dios. 4) Cuenca del Río Negro 5) la confluencia de Negro/Solimões. Además, en la literatura actual hay numerosas descripciones casuales y/o no cuantificadas de turba, lo que sugiere que probablemente el área de turberas de la cuenca de la Amazonía sea mayor a aquella cubierta por los estudios sistemáticos de las turberas. Las fuentes se enumeran en la Información Complementaria.

**Figura 2.** Ejemplos de los tres principales tipos de vegetación de turbera en la PMFB: (a) varillales hidromórficos, (b) turberas abiertas, (c) pantanos de palma.

**Figura 3.** Modelo de distribución del pantano de palma, varillales hidromórficos y turberas abiertas en la PMFB basada en datos de campo y de teledetección (Draper et al., 2014). Las fuentes de datos cartográficos se enumeran en la información complementaria.

**Figura 4.** Posibles amenazas al almacenamiento de carbono y biodiversidad en las turberas de la PMFB. Las fuentes se enumeran en la Información Complementaria.

**Figura 5.** Áreas protegidas y tierras tituladas en la PMFB. Las fuentes se enumeran en la Información Complementaria.
